# Methodological Complexities in Quantifying Rates of Fatal Opioid-Related Overdose

**DOI:** 10.1007/s40471-019-00201-9

**Published:** 2019-05-02

**Authors:** Svetla Slavova, Chris Delcher, Jeannine M. Buchanich, Terry L. Bunn, Bruce A. Goldberger, Julia F. Costich

**Affiliations:** 1 Department of Biostatistics, College of Public Health, University of Kentucky, Lexington, KY USA; 2 Kentucky Injury Prevention and Research Center, University of Kentucky, 333 Waller Ave, Suite 242, Lexington, KY 40504 USA; 3 Department of Pharmacy Practice and Science, Institute for Pharmaceutical Outcomes and Policy, College of Pharmacy, University of Kentucky, Lexington, KY USA; 4 Department of Biostatistics, Graduate School of Public Health, University of Pittsburgh, Pittsburgh, PA USA; 5 Department of Preventive Medicine and Environmental Health, College of Public Health, University of Kentucky, Lexington, KY USA; 6 Department of Pathology, Immunology and Laboratory Medicine, College of Medicine, University of Florida, Gainesville, FL, USA; 7 Department of Health Management and Policy, College of Public Health, University of Kentucky, Lexington, KY USA

**Keywords:** Opioid overdose, Drug poisoning, Drug overdose death, Postmortem toxicology, Enhanced opioid overdose surveillance, Prescription opioid, heroin, and fentanyl

## Abstract

**Purpose of Review:**

Effective responses to the US opioid overdose epidemic rely on accurate and timely drug overdose mortality data, which are generated from medicolegal death investigations (MDI) and certifications of overdose deaths. We identify nuances of MDI and certification of overdose deaths that can influence drug overdose mortality surveillance, as well as recent research, recommendations, and epidemiological tools for improved identification and quantification of specific drug involvement in overdose mortality.

**Recent Findings:**

Death certificates are the foundation of drug overdose mortality surveillance. Accordingly, counts and rates of specific drug involvement in overdose deaths are only as accurate as the drug listed on death certificates. Variation in systematic approaches or jurisdictional office policy in drug overdose death certification can lead to bias in mortality rate calculations. Recent research has examined statistical adjustments to improve underreported opioid involvement in overdose deaths. New cause-of-death natural language text analysis tools improve quantification of specific opioid overdose mortality rates. Enhanced opioid overdose surveillance, which combines death certificate data with other MDI-generated data, has the potential to improve understanding of factors and circumstances of opioid overdose mortality.

**Summary:**

The opioid overdose crisis has brought into focus some of the limitations of US MDI systems for drug overdose surveillance and has given rise to a sense of urgency regarding the pressing need for improvements in our MDI data for public health action and research. Epidemiologists can stimulate positive changes in MDI data quality by demonstrating the critical role of data in guiding public health and safety decisions and addressing the challenges of accurate and timely overdose mortality measures with stakeholders. Education, training, and resources specific to drug overdose surveillance and analysis will be essential as the nation’s overdose crisis continues to evolve.

## Introduction

**Fig. 1:**
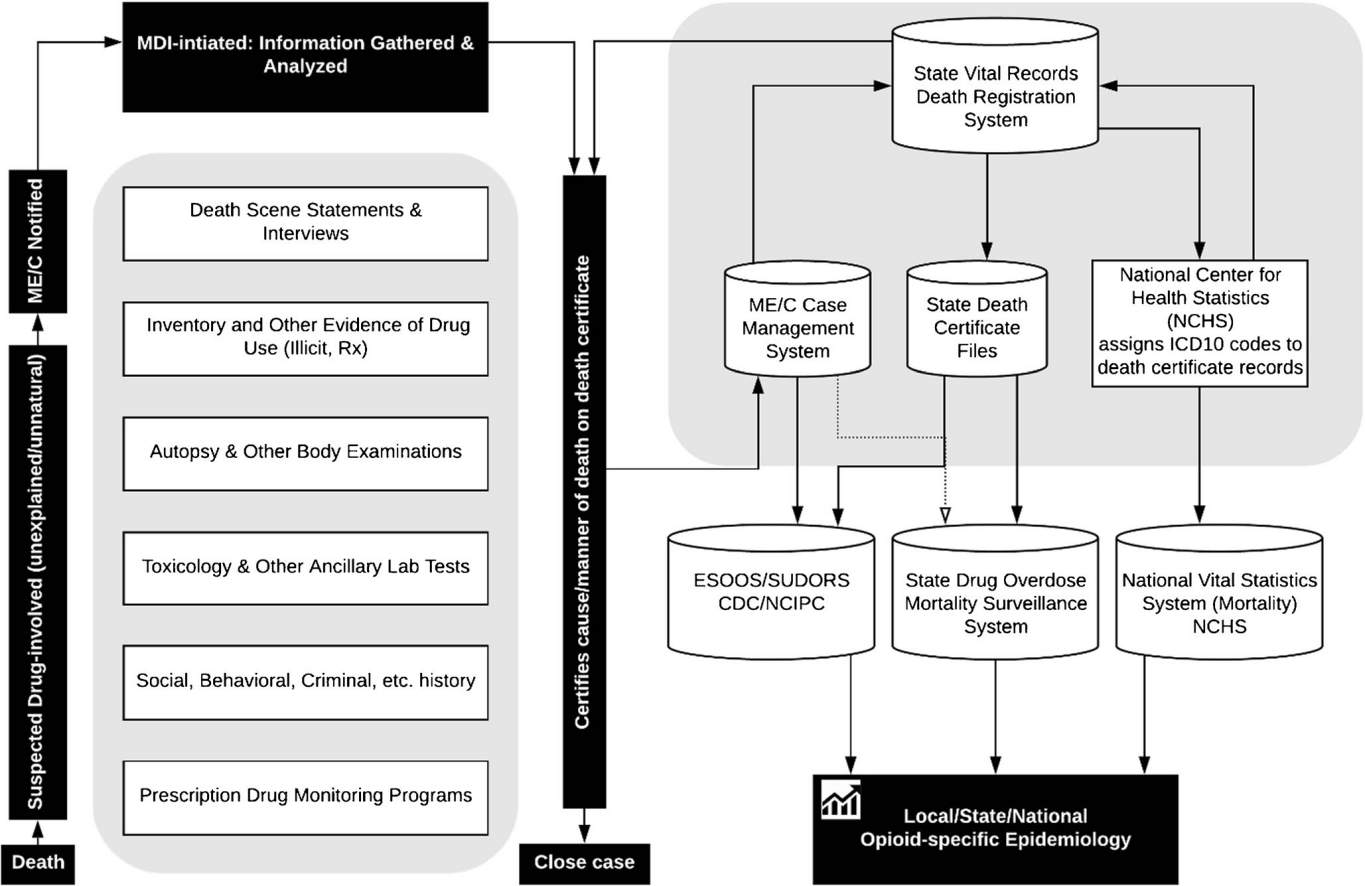
Medicolegal death investigation, certification, registration, surveillance, and epidemiology of drug overdose deaths. Abbreviations: Medicolegal Death Investigation (MDI), Medical Examiners and Coroners (ME/Cs), Enhanced State Opioid Overdose Surveillance (ESOOS), State Unintentional Drug Overdose Reporting System (SUDORS), Centers for Disease Control and Prevention (CDC), National Center for Injury Prevention and Control (NCIPC)

The increase in drug-related overdose deaths in the USA in the last two decades has been declared an epidemic, a crisis of historic scale, and a public health emergency [1–4]. Effective responses to the epidemic require accurate and timely drug overdose surveillance data [5–8]. Drug overdose mortality surveillance relies on the ability of the medicolegal death investigation (MDI) system to generate death certificates with complete and specific information on drugs that are responsible for or contributed to overdose deaths [9•, 10••, 11••]. However, lack of routinely performed comprehensive toxicology testing, analytical challenges to detection and quantification of novel synthetic opioids, and errors in death certificate completion can introduce bias in quantifying the involvement of specific drugs in drug overdose mortality [12••, 13, 14, 15••]. This review discusses the MDI system and certification of overdose deaths, death certificate coding using the International Classification of Diseases, Tenth Revision (ICD-10), approaches to drug overdose mortality data quality assessment, and epidemiological tools for improved identification of specific drugs to improve population estimates for opioid-related drug overdose mortality. Notably, the data used in drug overdose mortality surveillance is generated by multiple agencies and actors, with no single agency having quality control oversight of the entire process (Fig. 1). Epidemiologists who conduct drug overdose surveillance and researchers who work with drug overdose surveillance data must understand the processes underlying data generation and appreciate how their data and analytical results may be influenced by the evolving nature of these processes (Fig. 1).

## Investigation and Classification of Overdose Deaths

This section describes the processes relevant to drug overdose mortality surveillance. These processes are summarized in the left-hand side (“The MDI System and Certification of Opioid-Related Death”) and right-hand side (“Death Certificate ICD-10 Coding”) of Fig. 1.

### The MDI System and Certification of Opioid-Related Death

The goal of a MDI is to identify and certify the cause and manner of death. MDIs are initiated as required by state laws in cases of unexplained, sudden, and/or unnatural deaths (including suspected intentional or unintentional drug overdose deaths) [11••, 16–18]. The MDI system officers are medical examiners and/or coroners (ME/Cs). Medical examiners and coroners vary in their selection (appointed or elected) and professional credentialing [16–21].

There are defined steps in a competent MDI [22]. The ME/Cs’ primary responsibility in death registration is to complete the medical part of the death certificate for deaths over which they assumed jurisdiction [23]. Death certificates vary by jurisdiction but are all based on the US Standard Certificate of Death, 2003 revision [24]. The medical certification section describes the causal chain of events leading to death, from the initiating condition or injury (the “underlying cause of death” [UCOD]) to the immediate cause of death, describing also other significant conditions contributing to death, and the manner of death (e.g., natural, accident, suicide, homicide, undetermined) [9•, 23]. Multiple studies have reported that common ME/C errors in death certification can affect the accuracy of death certificate data for public health action [9•, 13, 25–30]. Hanzlick provides an excellent review on the important role of ME/Cs in generating MDI data for epidemiological research and the public health impact of MDIs in the USA [11••].

Recommendations for certifying opioid-related deaths are set out in three important papers [31••, 32••, 33••], as described below. The first of these, a position paper from the National Association of Medical Examiners (NAME), presented evidence-based recommendations for the investigation, diagnosis, and certification of deaths related to opioid drugs [31••]. The recommendations called for a complete death scene investigation, with a comprehensive toxicology panel and interpretation of toxicology results in the context of circumstances of death, listing all responsible substances by generic names on death certificates, and classification of deaths due to misuse or abuse of opioids without any apparent intent of self-harm as “accidents” [31••].

In the second paper, Goldberger et al. presented recommendations by a consensus panel convened by the Substance Abuse and Mental Health Services Administration (SAMHSA) predating the recent surge in opioid-related deaths [32••]. The panel provided recommendations on drug-related death scene investigation, a standard of practice for forensic toxicology, and guidance on documenting cause of death on death certificates, distinguishing between “drug-caused” and “drug-detected” deaths. The paper reiterated that the lack of uniform standards in classifying opioid drug-related deaths affects the quality of incidence and prevalence data and can be an obstacle for adequate public health and safety response [32••].

The third paper, from the Organization of Scientific Area Committees (OSAC) for Forensic Science MDI Subcommittee, reviewed current guidelines and proposed recommendations for drug-related MDI, indicating that “three initiatives are essential for informing timely and effective public health, law enforcement, and public policy responses to the opioid crisis.” These three initiatives are “adoption of standardized drug-related death investigation for ME/Cs, developing strategies for increased drug death surveillance by ME/Cs, and ensuring that ME/Cs have access to death certificates and other essential information for quality control and data analysis” [33••].

Forensic toxicology testing is essential for the accurate identification of involved drugs, including the novel psychoactive substances (NPS). However, the USA has no nationally-accepted best practices, standards, or guidelines for postmortem toxicology testing. Further, it is noteworthy that many ME/C jurisdictions do not test all suspected drug overdose deaths for NPS drugs, including fentanyl and fentanyl analogs [12••, 14, 31••, 32••, 34]. For example, the National Forensic Laboratory Information System report estimates that only 75% of the ME/Cs always request toxicology testing for opioids other than heroin or fentanyl, and only 51–75% always request toxicology testing for fentanyl and heroin [34].

Drugs considered “physiologically significant in causing death” should be listed individually (preferably with their generic names) in Part 1 of the Cause of Death portion of the death certificate, which describes the causal sequence leading to death [27, 32••]. Further, accurate certification requires that drugs that did not contribute to the cause of death should not be recorded on death certificates [13, 32••]. However, Gill pointed out that “in multi-drug intoxications, it usually is not possible to tease out an individual drug’s role” and thus “it is customary to include all the drugs (with concentrations greater than trace amounts) in the cause-of-death statement” [35•].

### Death Certificate ICD-10 Coding

After the death certificate is filed (electronically or as a paper copy) with the state office of vital statistics (OVS), the OVS electronically transmits a limited number of fields (including cause-of-death information) to the National Center for Health Statistics (NCHS) (Fig. 1). NCHS codes the cause-of-death text for epidemiological analysis using the World Health Organization (WHO) ICD-10 guidelines [36]. A single code is used for the UCOD, and up to 20 additional codes are assigned for contributing causes [37–39]. Statements such as “overdose of drug,” “toxic effect of drug,” “intoxication by drug,” “drug taken inadvertently,” “lethal amount of drug,” “wrong drug given in error,” or “wrong dose taken accidentally” are coded with an UCOD for drug poisoning, whether or not the drug was given in a treatment setting [38]. Based on the overdose intent and the type of drugs involved, a drug overdose death is coded with one UCOD code in the ICD-10 drug poisoning range: X40–X44 (accidental), X60–X64 (suicide), X85 (homicide), or Y10–Y14 (undetermined) [36]. Additionally, one or more codes in the range T36–T50 are used to describe the specific drugs/drug classes involved.

In the ICD-10 system, specific drugs are typically grouped into broad categories, making it difficult to identify deaths involving a particular drug. For example, oxycodone, codeine, hydrocodone, oxymorphone, and morphine are classified in the same category of natural and semisynthetic opioid analgesics (ICD-10 code T40.2) [36, 40•]. Identification of opioids is limited to the following ICD-10 codes: opium (T40.0); heroin (T40.1); other opioids (T40.2) [natural or semi-synthetic]; methadone (T40.3); other synthetic narcotics [excluding methadone] (T40.4); or other and unspecified narcotics (T40.6) [e.g., “opioid,” “opiate”]. Of special interest is T50.9 (other and unspecified drugs). When a drug overdose death record includes T50.9 as the only contributing drug code in the range T36–T50, it typically means that no drug name or drug class was listed on the death certificate [10••, 15••, 41••, 42••]. In 2012, the Safe States Injury Surveillance Workgroup on Poisoning released Consensus Recommendations for Poisoning Surveillance, providing a poisoning classification matrix for ICD-10-coded mortality data [43•]. This matrix can help researchers identify the UCOD and contributory causes for analyses of opioid- and other drug-related deaths.

## Epidemiologic Analysis of Drug Overdose Death Data

Timely and accurate analysis of overdose mortality data is complicated by diverse factors, including (but by no means limited to) the lack of specific drug information, regional variations in undetermined intent coding, MDI delays in finalizing death certificates for suspected drug overdose deaths, and the rapidly evolving nature of the opioid overdose crisis itself. This section will discuss these issues and some of the techniques that have been used to mitigate their impact on the statistics generated from drug overdose mortality data. The reader should bear in mind that this is a rapidly evolving topic and new statistical techniques and approaches may be developed in the near future.

### Methodologic Considerations for the Analysis of ICD-10-Coded Death Certificate Data

Counts and rates of overdose deaths involving specific drugs are only as accurate as the drugs listed on death certificates. If drugs are not listed because of a certifier’s systematic approach or jurisdictional office policy, rate quantifications could be severely biased. Warner et al. showed that states with centralized medical examiner systems had on average higher percentage completeness on listed drugs (92%), compared with states with decentralized systems (medical examiner (71%), hybrid ME/C (73%), or coroner (62%)) [10••].

Slavova et al. provided examples of misleading rankings of jurisdictions by age-adjusted opioid analgesic overdose rates when some jurisdictions reported disproportionately high rates of overdose deaths associated with “other and unspecified drugs” or “other and unspecified narcotics” [15••]. The paper presented recommendations from the Council of State and Territorial Epidemiologists Overdose Subcommittee (CSTE ODS) that “epidemiologists and other public health practitioners need to be aware of the quality and limitations of the death certificate data in their jurisdiction, evaluate when possible the level of completeness and accuracy of the ICD-10 codes, and interpret the reported counts and rates with caution when the proportion of deaths with unspecified drugs is considerable.” The paper also recommended that jurisdictional comparisons should be based on total drug overdose death rates, while noting that “trends in jurisdictional rates for specific drug types remain useful as long as the degree of specification of drugs does not vary markedly from year to year” [15••].

A cautionary example illustrating the effects of an abrupt change in degree of drug specification comes from South Carolina (SC). The reported age-adjusted prescription opioid-related poisoning (T40.2–T40.4) mortality rate in SC was 4.7/100,000 in 2013, rising to 9.3/100,000 in 2014 [44]. The twofold increase could be interpreted mistakenly as a sudden worsening in opioid overdose deaths in SC. However, in reality, it primarily reflected an impressive increase in the percentage of drug poisoning death certificates that listed specific contributing drugs (57.7% in 2013; 94.4% in 2014), attributable to the January 2014 implementation of a SC Office of Vital Statistics process to collect specific drug names for all deaths (Daniela Nitcheva, Biostatistics Division Director, SC OVS, personal correspondence). An abrupt change could also be a result of new drug panels in toxicology testing. For example, the introduction of routine gabapentin testing in Kentucky in July 2014 resulted in a significant increase in the prevalence of gabapentin among drug overdose decedents, from 2.9% in 2013 to 17% in 2014 [45•]. These examples illustrate abrupt procedural changes that generated dramatic upward shifts in reported rates. We caution that gradual improvements in procedures over time generate smaller annual changes in overdose mortality rates, and therefore are harder to detect.

Challenges in determining the manner of drug-related deaths have implications for public health research and policy [46]. Variation among MDI jurisdictions in classifying drug poisoning deaths as “undetermined intent” versus “unintentional” or “suicide” affects jurisdictional comparison of drug poisoning death rates by intent [47, 48]. Warner et al. showed that 85% of the poisoning deaths in Maryland and more than 40% in Utah were classified as undetermined intent, while the national average was 8% [10••]. To address these variations, the NAME position paper recommends classifying deaths from misuse or abuse of opioids that lack any apparent intent of self-harm as “accidents,” reserving “undetermined” as the manner for the *rare* cases in which evidence exists to support more than one possible intent determination [31••]. As of 2016, the percentage of drug poisoning deaths with undermined intent still varied widely across states, from 1% in Connecticut and Massachusetts to 68% in Maryland [44]. The variation in manner-of-death determination among states may be influenced by ME/C office policies that are not aligned with recommendations provided by professional associations [31••] and this variation complicates both between-state and temporal comparisons.

Delay in finalizing death certificates for suspected drug overdose deaths is a source of undercounting in provisional mortality data. ICD-10 code R99 (unspecified causes of mortality) is typically used as a UCOD for injury-related deaths until the investigative process is complete. An NCHS study showed an inverse relationship between the percentage of deaths with UCOD R99 and those coded as drug poisonings [49•].

There are also methodologic complexities related to the rapidly evolving nature of the opioid overdose crisis. The rapidly escalating involvement of illicitly manufactured fentanyl (IMF) in US poisoning deaths has required reexamination of surveillance definitions for prescription opioid involvement. Historically, the Centers for Disease Control and Prevention (CDC) used the ICD-10 codes T40.2–T40.4 to identify “prescription opioid overdose deaths.” Seth et al. proposed a more conservative definition, excluding T40.4 because of “increasing evidence that recent deaths involving synthetic opioids are likely a result of IMF” [50]. The authors argued that this measure was a more realistic count of the involvement of pharmaceutically produced (versus illicitly manufactured) opioids.

It is important to note, however, that IMF began flooding the US markets in 2013–2014, with significant presence in Northeastern and Southern states [51]. Opioid analgesics overdose deaths are widespread across the USA, whereas fentanyl overdose deaths are predominantly located in the northeastern USA [52]. In 2016, the percentage of overdose deaths coded as T40.4 among overdose deaths with contributing codes T40.2–T40.4 varied from less than 20% in Nevada and Utah to more than 80% in New Hampshire and Massachusetts [44]. Therefore, epidemiologists may need to consider different definitions to improve measurement of prescription opioid involvement in specific populations at different times.

### Identifying Specific Opioids by Cause-of-Death Text Analysis

A serious limitation of ICD-10-coded death certificate data is that it does not allow identification of most specific opioids (e.g., oxycodone, fentanyl, fentanyl analogs). The cause-of-death text on death certificates can be used to help overcome this limitation by supporting calculation of age-adjusted overdose rates for most frequently listed drug names [53•, 54]. It is important to note that this technique cannot be applied to the approximately 20% of US overdose death certificates that do not identify any specific drugs.

A novel cause-of-death text search tool was developed by the NCHS and the US Food and Drug Administration (FDA), allowing identification of drugs mentioned as well as contextual information [55••]. Warner et al. used the tool to identify drugs most frequently involved in drug overdose deaths in the USA, 2010–2014 [40•]. It would greatly enhance national and state mortality surveillance and research if the NCHS were to develop a mechanism to add new variables to ICD-10-coded electronic death certificate records to identify contributing drugs by their generic names. However, as noted above, this approach still would not compensate for the opioid involvement undercounting that is attributable to the lack of any specific drug identification on about 20% of overdose death certificates.

### Statistical Adjustment for Undercounting

Recent research has examined the effect of incomplete death certificate data on the estimated magnitude of the US opioid epidemic. Buchanich et al. reallocated a proportion of the unintentional unspecified drug overdose deaths to the unintentional opioid-related overdose death category, by state and year. The underlying assumption was that the proportion of unspecified drug unintentional overdose deaths that might be attributed to opioids would be the same as the proportion of opioid-related overdose deaths among all unintentional overdose deaths with specified drugs [41••]. The authors concluded that, between 1999 and 2015, some 70,000 opioid-related unintentional overdose deaths could have been unaccounted for due to incomplete cause-of-death information.

Ruhm described statistical adjustment methods to account for underreported involvement of specific drugs in overdose deaths [56]. Applying a statistical model-based approach, he calculated a 2015 US opioid-involved drug poisoning mortality rate estimate (12.46/100,000) that was 21% higher than the reported rate (10.31/100,000) [57]. Ruhm also calculated adjusted opioid- and heroin-involved drug poisoning mortality rates by state, 2008–2014, showing wide variations in the percentage change between reported and adjusted mortality rates that reflected variations in proportions of overdose deaths with unspecified drugs [58••].

The assumptions made by Buchanich et al. and Ruhm are reasonable but difficult to verify. Predictive models may be improved by including explanatory variables for key characteristics of MDI jurisdictions associated with consistency, completeness, and accuracy of drug-related information listed on death certificates (e.g., ME/C jurisdictional type, toxicology testing protocols), but such information is not readily available.

Timeliness of drug overdose death certification could be another source of undercounting in provisional mortality data. An NCHS report found that the lag time between the week of death and the week when information on the death certificate became available for production of provisional estimates for drug overdose deaths was much longer than for natural deaths or suicides, usually due to delays in forensic toxicology analysis [49•]. The NCHS used regression models to predict completeness of provisional data relative to final data and to report estimated provisional number of drug overdose deaths [59•, 60].

### Enhanced Drug Overdose Mortality Surveillance

Ongoing research is demonstrating the value of enhancing the epidemiological analysis of death certificate data with other MDI-generated data. For example, epidemiologists can utilize toxicology data to improve estimates of heroin involvement in overdose deaths where the definitive heroin metabolite, 6-acetylmorphine (6-AM), was not detected by toxicological analyses, but other data indicate heroin ingestion. Heroin-specific analytes often go undetected due to the rapid metabolism of heroin to 6-AM, and the subsequent metabolism of 6-AM to morphine [61]. However, codeine is often present in heroin as an impurity. Recent research has demonstrated that a morphine-to-codeine concentration ratio greater than 1 in toxicological data is a strong and probable indicator of heroin involvement and can be used to adjust the heroin-related overdose count/rate estimations [61–68]. Roxburgh et al. provided flowcharts for distinguishing between morphine, codeine, and heroin deaths [69]. Positive toxicology for 4-ANPP, a precursor to the IMF, and a metabolite of several fentanyl analogs can be used to identify involvement of IMF or fentanyl analogs [70–72].

Epidemiological analysis that supports rapid response planning can benefit from identification of drug overdoses resulting in rapid deaths. Because of its short half-life, the presence of heroin metabolites can be interpreted as a proxy for short survival time [73, 74]. Lack of detectable concentrations of norfentanyl (a fentanyl metabolite) could indicate a rapid death following fentanyl administration [75, 76].

The role of benzodiazepines and other non-opioid drugs in multi-drug toxicity deaths is not well understood or documented. A Kentucky study reported that benzodiazepines were less likely to be listed as contributing drugs on death certificates (67% for alprazolam, less than 40% for clonazepam and diazepam) compared with fentanyl (89%), heroin (88%), methadone (81%), or cocaine (77%) [62]. Further analysis of toxicological results could provide information on the presence of benzodiazepines in concentrations sufficient to have caused or contributed to death in each case.

Lack of routine testing for specific analytes in some jurisdictions affects the frequency of detected substances and impedes jurisdictional comparisons. For example, in a multi-jurisdictional study on gabapentin involvement in drug overdose deaths, the region with the lowest gabapentin prevalence was also the only one that did not test for gabapentin in every potential drug overdose case [77].Reporting the type of postmortem toxicology testing (screening vs. confirmatory; routine vs. request-only) should become a standard practice, especially for multi-jurisdictional studies. Implementing such a reporting standard will require communication among drug overdose surveillance epidemiologists, ME/Cs, and toxicology labs [42••].

There is ample evidence that supplementing death certificate data with other MDI-generated data sources can improve our understanding of drug overdose epidemiology [62, 78–82]. A surveillance system requires continuous and systematic data collection, ongoing analysis, and interpretation of outcome-specific data to inform planning, implementation, and evaluation of drug overdose prevention programs and policies [11••, 83]. While drug overdose surveillance systems based solely on death certificate data can provide important information on trends in drug overdose, they cannot address issues such as drug diversion or risk factors for overdose, and their timeliness depends on the time to final determination of death [49•, 59•, 84].

As described by Hargrove et al., Kentucky developed an enhanced multi-source drug overdose fatality surveillance system in 2014–2015, generating timely new data and on-going analysis that had a significant impact on state policy (e.g., contributing to the scheduling of gabapentin as a controlled substance) [84•].

In 2016–2017, CDC supported 32 states to establish an Enhanced State Opioid Overdose Surveillance (ESOOS) program [8]. One of the program’s goals was to develop state capacity for timely and comprehensive data collection and reporting of fatal opioid overdoses. At least 60% of September 2017 supplemental ESOOS funding was mandated to support ME/Cs, including for comprehensive toxicology testing. Funded ESOOS states enter data into the State Unintentional Drug Overdose Reporting System (SUDORS). Although still limited to unintentional opioid overdose deaths, the system allows comparison among funded states, using death certificate data supplemented with ME/C data “previously unavailable across states” and “provides unique insights into specific substances and circumstances associated with overdoses” [85, 86].

## Death Certificates Remain the Core of Population-Based Drug Overdose Mortality Surveillance

Death certificates have been, and will remain, the core of national and state drug overdose mortality surveillance. Drug overdose surveillance based on ICD-coded death certificate data allows standardized comparisons of state, national, and international drug overdose mortality trends. Death certificate data are available for all geographic and demographic groups. The ICD coding performed at the NCHS ensures standardized classification of the cause-of-death information for epidemiological analysis. The grouping of specific drugs in ICD-10-coded categories is an obstacle for analysis of specific drug involvement in overdose deaths, but even this limitation can be overcome with wider adoption of the cause-of-death text analysis tools.

As ME/Cs have come to appreciate the importance of MDI information for public health surveillance that supports policy and program decisions, they have made significant improvements in death certificate reporting of drugs involved in overdose deaths. Hedegaard et al. reported that the percentage of drug overdose death certificates that identified specific drugs or drug classes has “increased each year (75% in 2011, 76% in 2012, 78% in 2013, 81% in 2014, 83% in 2015, and 85% in 2016)” [87•], and asked whether improved reporting influenced observed trends in drug overdose deaths for specific drugs [87•]. To address this question, the authors conducted an adjustment analysis (a description of the methodology and results of the adjustment analysis are provided in the paper’s technical notes) and described factors influencing the quality and completeness of specific drug information on overdose death certificates [87•].

More specific and timely identification of drugs involved in drug overdose deaths requires adequate MDI capacity, infrastructure, and trained personnel. Regarding MDI capacity and infrastructure, we note that the 2017 ME/C Office Survey reported that only 32% of the responding ME/Cs had computerized, networked systems [34]. Currently, many initiatives are under way to address these issues. For example:

I. The National Research Council of the National Academies provided in-depth analysis of obstacles to quality control and quality assurance in MDIs and offered “a path forward” recommendations [88].
II. A MDI Federal Interagency Working Group (MDI-WG) coordinates federal initiatives to strengthen the MDI system and support ME/C services [89]. The MDI-WG proposed steps towards a modern, professional, and efficient MDI system that can provide accurate, comparable, and timely data to policymakers, researchers, and public health and safety officials [90].

III. The National Science and Technology Council’s Fast-Track Action Committee on Strengthening the MDI System (FTAC-SMDIS) has provided strategic policy recommendations on the role of the federal government in enhancing MDI data infrastructure and fostering standards to support high-quality postmortem toxicology testing, among others [7].

IV. The MDI Subcommittee, Organization of Scientific Area Committees for Forensic Science, released a strategy statement for ME/C drug related death investigations [91]. The statement also points to the role of epidemiologists and statisticians in using MDI data to inform policy and programs, and the need for developing local, regional, and national incident surveillance systems to improve outbreak detection and rapid response.

V. The CSTE Overdose Subcommittee (CSTE ODS) was established in 2012 in response to the growing prescription drug overdose epidemic in the USA. The CSTE ODS has been working on raising the CSTE membership awareness on methodological issues and framework for analyzing drug overdose death data and building epidemiological capacity for drug overdose surveillance [15••, 42••, 53•, 92].
VI. The Association of State and Territorial Health Officials, with input from MDI professionals and surveillance experts (including epidemiologists, statisticians, and Vital Statistics state registrars), released a report on key strategies, priorities, and feasible action areas for improving completeness and drug specificity on drug overdose death certificates [93].
VII. CDC’s strategic plan for improving public health surveillance includes expanding the electronic death reporting system into a system capable of supporting near real-time mortality surveillance, allowing faster cause-of-death notification, and reporting of national and state provisional counts of drug overdose deaths [60, 94, 95].

## Conclusions

The opioid overdose crisis has given rise to a sense of urgency regarding the need for improvement in the accuracy and timeliness of current MDI data. However, the fragmented nature of current MDI systems makes it unlikely that implementation of uniform national standards will be feasible in the near future. Epidemiologists involved in drug overdose fatality surveillance and research can play important roles in promoting positive changes in MDI data quality by demonstrating the critical role of the data in guiding public health and safety decisions and addressing the challenges for accurate and timely overdose mortality measures with stakeholders (e.g., ME/Cs, vital statistics registrars). It is also critical that researchers who analyze drug overdose mortality data appreciate the limitations of the current MDI systems for drug overdose surveillance. Support for epidemiologists and public health researchers in the form of education, training, and resources specific to drug overdose surveillance and analysis will be essential as the nation’s overdose crisis continues to evolve.
